# RBP-TSTL is a two-stage transfer learning framework for genome-scale prediction of RNA-binding proteins

**DOI:** 10.1093/bib/bbac215

**Published:** 2022-06-02

**Authors:** Xinxin Peng, Xiaoyu Wang, Yuming Guo, Zongyuan Ge, Fuyi Li, Xin Gao, Jiangning Song

**Affiliations:** Biomedicine Discovery Institute and Department of Biochemistry and Molecular Biology, Monash University, Melbourne, Victoria 3800, Australia; Monash Data Futures Institute, Monash University, Melbourne, Victoria 3800, Australia; Biomedicine Discovery Institute and Department of Biochemistry and Molecular Biology, Monash University, Melbourne, Victoria 3800, Australia; Monash Data Futures Institute, Monash University, Melbourne, Victoria 3800, Australia; Department of Epidemiology and Preventive Medicine, School of Public Health and Preventive Medicine, Monash University, Melbourne, Victoria 3004, Australia; Monash e-Research Centre and Faculty of Engineering, Monash University, Melbourne, VIC 3800, Australia; Biomedicine Discovery Institute and Department of Biochemistry and Molecular Biology, Monash University, Melbourne, Victoria 3800, Australia; Department of Microbiology and Immunology, The Peter Doherty Institute for Infection and Immunity, The University of Melbourne, 792 Elizabeth Street, Melbourne, Victoria 3000, Australia; College of Information Engineering, Northwest A&F University, Yangling, 712100, China; Computer Science Program, Computer, Electrical and Mathematical Sciences and Engineering Division, King Abdullah University of Science and Technology (KAUST), Thuwal 23955-6900, Kingdom of Saudi Arabia; KAUST Computational Bioscience Research Center, King Abdullah University of Science and Technology; Biomedicine Discovery Institute and Department of Biochemistry and Molecular Biology, Monash University, Melbourne, Victoria 3800, Australia; Monash Data Futures Institute, Monash University, Melbourne, Victoria 3800, Australia

**Keywords:** RNA binding proteins, knowledge transfer learning, deep learning, pre-trained language model, sequence analysis

## Abstract

RNA binding proteins (RBPs) are critical for the post-transcriptional control of RNAs and play vital roles in a myriad of biological processes, such as RNA localization and gene regulation. Therefore, computational methods that are capable of accurately identifying RBPs are highly desirable and have important implications for biomedical and biotechnological applications. Here, we propose a two-stage deep transfer learning-based framework, termed RBP-TSTL, for accurate prediction of RBPs. In the first stage, the knowledge from the self-supervised pre-trained model was extracted as feature embeddings and used to represent the protein sequences, while in the second stage, a customized deep learning model was initialized based on an annotated pre-training RBPs dataset before being fine-tuned on each corresponding target species dataset. This two-stage transfer learning framework can enable the RBP-TSTL model to be effectively trained to learn and improve the prediction performance. Extensive performance benchmarking of the RBP-TSTL models trained using the features generated by the self-supervised pre-trained model and other models trained using hand-crafting encoding features demonstrated the effectiveness of the proposed two-stage knowledge transfer strategy based on the self-supervised pre-trained models. Using the best-performing RBP-TSTL models, we further conducted genome-scale RBP predictions for *Homo sapiens*, *Arabidopsis thaliana*, *Escherichia coli*, and *Salmonella* and established a computational compendium containing all the predicted putative RBPs candidates. We anticipate that the proposed RBP-TSTL approach will be explored as a useful tool for the characterization of RNA-binding proteins and exploration of their sequence–structure–function relationships.

## Introduction

RNA-binding proteins (RBPs) form ribonucleoprotein complexes when binding with RNAs. They play crucial roles in the post-transcriptional regulation of RNAs and are potential biological markers for the cancer diagnosis [1–5]. The wet-lab experimental methods, such as RNA Interactome Capture (RIC) [6], can accurately identify RBPs. However, these approaches are time-consuming and cost-intensive, which are not suitable for high-throughput identification of RBPs [7]. In addition, RIC can only work on RBPs with functional poly(A) tails on transcripts and efficient incorporation of crosslink-enhancing artificial nucleotides [8], thereby limiting its application to RBPs in prokaryotes. In this context, computational approaches for *in silico* high-throughput prediction of RBPs can help guide hypothesis-driven experimental studies of RBPs. To date, a variety of computational methods have been developed for this purpose. These methods can be generally classified into two major groups: template-based and machine learning-based.

The first group of methods identifies RBPs by calculating the similarity score between a query protein and the template RBPs or RNA-binding domains (RBDs). Methods in this category include SPalign [9], SPOT-stru [10], SPOT-seq [10], SPOT-Seq-RNA [11], and APRICOT [12]. The similarity score is typically calculated based on the amino acid substitution matrix combined with sequence-derived features, such as dipeptides, tripeptides, and physicochemical properties. However, on one hand, the predefined RBDs cannot be found in almost half of the experimentally identified RBPs [13, 14]. On the other hand, proteins with the presence of the RBDs may not necessarily correspond to RBPs [15]. In these two cases, the template-based methods would be ineffective.

The second group of methods applies machine learning techniques to train models using annotated training datasets containing both RBPs and non-RBPs. These machine learning-based models rely on the representation of useful features and the construction of prediction models based on the given positive and negative samples. Different types of features and machine learning methods have been explored [16–37]. Machine learning methods have gained popularity in recent years due to their attractive advantage in effectively dealing with the high-dimensional features derived from sequences or structures and modeling the sequence–structure–function relationship of RBPs. Among these methods, NAbind [30], BindUP [22], and NucleicNet [38] are three representative methods that leverage the prior 3D structure information to improve the predictive performance. In contrast, the majority of the existing methods rely on the use of sequence information to train the prediction model, which is more accessible compared with 3D structure data. A summary of currently available computational methods is provided in Table 1.

**Table 1:** A list of the reviewed methods for RNA-binding proteins prediction

Predictor category	Method	Means of classification	Properties	Dataset for training
Structure-based predictors	NAbind [30]	SVM–Gist	Electrostatic properties	76 RBPs, and 246 non-RBPs across species
	BindUP [22]	SVM–Gist	The same as NAbind	90 DNA-binding, 60 RNA-binding, and 300 non-NA-binding chains across species
Sequence-based predictors	SPOT-seq-RNA [11]	Template-based	Sequence-structure match and binding affinity	1052 RNA-binding domains and chains and 5766 non-RNA-binding chains across species
	RNApred [28]	SVM–SVM light	Amino acid composition, predicted binding residues from prior knowledge	377 RBPs and 377 non-RBPs across species
	RBPPred [21]	SVM	Evolutionary information, amino acid composition and physicochemical properties	2780 RBPs and 7093 non-RBPs across species
	Deep-RBPPred [20]	CNN	Amino acid composition and physicochemical properties	Same as RBPPred
	TriPepSVM [19]	string kernel SVM	Tri-peptide frequency	1812 known RBPs for *H. sapiens*, 306 for *Salmonella* and 512 for *E. coli*, 12,038 non-RNA-binding proteins for *H. sapiens*, 1415 for *Salmonella*, and 3783 non-RNA-binders for *E. coli*
	ProNA2020 [18]	SVM, Random Forests (RF), and Neural Networks (NN)	Evolutionary information and embeddings from Word2Vec model	263 RBPs and 555 non-RBPs across species
	RBPro [17]	RF	Amino acid composition and evolutionary information	2780 RBPs and 7093 non-RBPs across species
	IDRBP-PPCT [16]	RF	Evolutionary information	2945 RBPs and 4175 non-RBPs across species
	AIRBP [34]	an ensemble-based machine learning model	Evolutionary information, physicochemical properties, and disordered properties	Same as RBPPred
	PreRBP-TL [36]	Motif CNN	Evolutionary information	2982 RBPs and 48,352 non-RBPs for general pre-training dataset across species, 1296 RBPs and 9427 non-RBPs for *H. sapiens*, 480 RBPs and 6269 non-RBPs for *A. thaliana*, 389 RBPs and 3132 non-RBPs for *E. coli*, and 228 RBPs and 1230 non-RBPs for *Salmonella*

*Note*: Only the available approaches are listed in the Table.

Despite the outstanding performance achieved by these machine learning methods, it comes at the high cost of time-consuming calculation and extraction of features such as the position-specific scoring matrix (PSSM) generated by the PSI-BLAST [39] search. Furthermore, in some cases, physicochemical features cannot thoroughly represent the specific properties of proteins. For example, the two sequences ‘HLTHAQSTLDAK’ and ‘KHLTHAQSTLDA’ have the same amino acid compositions but represent different functionalities. In addition, for the methods that use the 3D structure information as the input, there only exists a small portion of samples that have available 3D structure data, which will limit their further application. Therefore, methods that can utilize the sequence information to accurately identify RBPs are highly desirable.

More recently, the transformer model and its variants [40] pre-trained on a massive amount of proteins sequences using self-supervision approaches have demonstrated a significant potential for harnessing the power of big data. Existing studies showed that the pre-trained models could substantially improve the predictive performance on various tasks [41–47]; however, this has not been systematically examined on the task of RBPs prediction. In this study, we develop a new approach called RBP-TSTL by integrating the feature embeddings generated by a self-supervised pre-trained model with the knowledge transferred from the annotated pre-training RBPs dataset. Benchmark experiments on the independent test datasets and additional validation datasets illustrate that RBP-TSTL models outperform state-of-the-art methods across four different species including *Homo sapiens, Arabidopsis thaliana, Escherichia coli*, and *Salmonella*. Moreover, we further perform genome-scale prediction of species-specific RBPs by applying the optimized model and accordingly provide the results as a computational compendium, which are publicly available at https://github.com/Xinxinatg/RBP-TSTL/.

## Materials and methods

### Datasets

#### Benchmark and annotated pre-training datasets

In this study, the benchmark and annotated pre-training datasets were taken from [36], which encompassed four species-specific datasets of RBPs, including *H. sapiens, A. thaliana, E. coli*, and *Salmonella*, as well as those in all other species available in the Swiss-Prot [48] as the annotated pre-training dataset. The positive samples were retrieved using the QuickGO API [49] from UniProtKB [50], while the negative ones were collected by removing annotated nucleotide-binding proteins and proteins containing any annotated or potential RBD identified in Pfam database [51]. The ratio of positive samples to negative samples was approximately 1:10. Notably, each dataset contains a training set, a validation set, and an independent test set with the percentage of 81, 9, and 10%, respectively. The training set was used to train and fine-tune the prediction model, the validation set to optimize the model, and the independent test set to evaluate and compare the performance of our method and other existing methods. To avoid potential bias and performance overestimation, the redundancy of the sequences in the training, validation, and those in the independent test datasets of each species was removed at the similarity identity threshold of 25% [21, 36] using CD-HIT [52]. Finally, we obtained 51 334, 12 103, 7907, 3951, and 1631 proteins in the annotated pre-training, *H. sapiens*, *A. thaliana*, *E. coli,* and *Salmonella* datasets, respectively.

Recent studies [19, 36] have shown that RBPs have species-specific characteristics, which are against the presumption that the properties of RNA-binding proteins are purely molecular-based and shared across all different species. For example, the tripeptides critical to the identification of RBPs are often present in *H. sapiens* but not in *Salmonella* and *E. coli* [19]. Furthermore, glutamate, serine, and proline are more abundant in the sequences of *H. sapiens* RBP, which is in contrast to the prevalence of alanine and arginine residues in the sequences of *E. coli* RBPs [36]*.* Due to these reasons, it is necessary to use species-specific datasets to capture the characteristics of RBPs.

#### Additional validation sets of experimentally identified RBPs

The additional validation sets were retrieved from the PreRBP-TL repository. According to the previous work of TriPepSVM [19], the additional validation sets were generated by removing sequences on which RBP-TSTL was trained from all the experimentally identified RBPs. In the current work, the RBPs collected from surveys for additional validation in TriPepSVM and PreRBP-TL were discarded as these RBPs were determined by the existence of the RBDs [14, 51]. As aforementioned in Introduction section, predefined RNA-binding-related homology domains are not present in almost half of the identified RBPs. Finally, the additional validation sets contained 110 proteins (denoted as ‘Set1’) collected from the study of Castello, Horos [53], and 108 proteins (denoted as ‘Set2’) from the study of Van Nostrand *et al.* [14], respectively.

### Framework of RBP-TSTL

**Figure 1:**
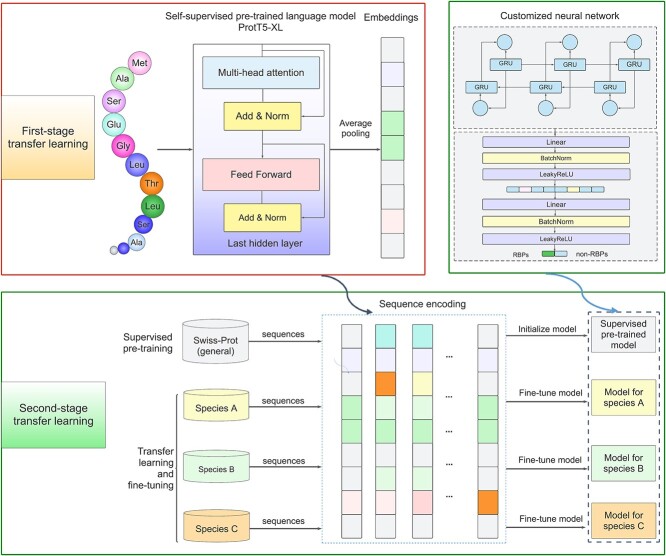
The model architecture of RBP-TSTL to predict RBPs. The RBP-TSTL model combined the ProtT5-XL model, a six-layer GRU, and a linear network section as the major architecture. The input of the model is protein sequences of arbitrary length composed of 20 standard amino acids. ProtT5-XL extracted sequence embeddings for first stage knowledge transfer, while GRU layers and discriminator generated the final classification result during the second stage knowledge transfer.

Inspired by the recent progress of transfer learning to address protein-nucleotide interaction problems [36, 54–56], in this study, we developed a two-stage transfer learning strategy for the genome-scale prediction of RBPs. The first stage of RBP-TSTL involved use of the knowledge transferred from a self-supervised pre-trained model named ProtT5-XL [43]. At the second stage, the knowledge in the annotated pre-training dataset for the RBPs prediction was transferred to each of the four target species. More specifically, the sequence embeddings were generated by the self-supervised pre-trained model ProtT5-XL [43] for both the annotated pre-training RBPs dataset and species-specific datasets. Subsequently, the embeddings for the annotated pre-training RBPs dataset were used to initialize our deep learning model prior to being fine-tuned on each species-specific dataset. In this way, the knowledge from the self-supervised pre-trained model and the annotated pre-training RBPs dataset could be transferred to address the RBPs prediction task for the target species. The methodological details of our RBP-TSTL method are illustrated in Fig. 1.

### Model training

#### Components of the deep learning model used in RBP-TSTL

The deep learning model at the second stage of transfer learning comprised six layers of Gated Recurrent Unit (GRU) [57] and a discriminator which was implemented using a linear network with the batch-normalization layer [58] and the Leaky ReLU [59] as the activation function.

GRU is a gating mechanism that uses each recurrent unit to capture the dependencies of different time scales adaptively. GRU has two gates, namely the update gate (${z}_t$) and reset gate (${r}_t$), as well as one state ${h}_t$. Each layer in the GRU implements the following computation:

(1) \begin{equation*} {\mathrm{z}}_{\mathrm{t}}=\sigma \left({\mathrm{W}}_{\mathrm{z}}\bullet \left[{\mathrm{h}}_{\mathrm{t}-1},{\mathrm{x}}_{\mathrm{t}}\right]+{\mathrm{b}}_{\mathrm{z}}\right) \end{equation*}

(2) \begin{equation*} {\mathrm{r}}_{\mathrm{t}}=\sigma \left({\mathrm{W}}_{\mathrm{r}}\bullet \left[{\mathrm{h}}_{\mathrm{t}-1},{\mathrm{x}}_{\mathrm{t}}\right]+{\mathrm{b}}_{\mathrm{r}}\right) \end{equation*}

(3) \begin{equation*} {\overset{\sim }{\mathrm{h}}}_{\mathrm{t}}=\tanh \left(\mathrm{W}\bullet \left[{\mathrm{h}}_{\mathrm{t}-1},{\mathrm{r}}_{\mathrm{t}}\right]+\mathrm{b}\right) \end{equation*}

(4) \begin{equation*} \mathrm{h}=\left(1-{\mathrm{z}}_{\mathrm{t}}\right)\bullet{\mathrm{h}}_{\mathrm{t}-1}+{\mathrm{z}}_{\mathrm{t}}\bullet{\overset{\sim }{\mathrm{h}}}_{\mathrm{t}}, \end{equation*}

where ${W}_z$, ${W}_r$, and $W$ denote the weights of GRU, ${b}_z$, and ${b}_r$, respectively, and $b$ are the bias vectors.

#### Weighted binary cross-entropy

The weighted binary cross-entropy was adopted to counter the imbalance of the datasets. The loss function is formulated as follows:

(5) \begin{equation*} J=-\frac{1}{N}\sum_{i=1}^N\left[w\times{y}_i\mathit{\log}\left({p}_i\right)+w\times \left(1-{y}_i\right)\mathit{\log}\left(1-{p}_i\right)\right] \end{equation*}

(6) \begin{equation*} \mathrm{w}=\frac{1-\mathrm{pos}\_\mathrm{ratio}}{\mathrm{pos}\_\mathrm{ratio}}, \end{equation*}

where ${y}_i$ is the label of the *i*th protein, ${p}_i$ is the logit output from the discriminator layer for the *i*th protein being RBP, while $pos\_ ratio$ represents the percentage of RBPs in the analyzed dataset.

#### Experimental settings

RBP-TSTL was implemented using PyTorch (version 1.7.1), an open-source machine learning framework [60]. The procedures for training the neural networks are briefly described as follows: we used optimization-ADAM [61] which is an SGD-based algorithm as the optimizer and set the number of GRU layers to 6. To avoid overfitting, we adopted the early-stop strategy based on the performance of the validation sets and used the ReduceLROnPlateau scheduler [62] to adaptively reduce the learning rate when the metric was not improving. A detailed description of the tuned hyperparameter values can be found in Supplementary Table S1 (see Supplementary Data available online at https://academic.oup.com/bib).

### Performance assessment

Four major performance evaluation metrics were adopted to gauge the performance of different methods according to the previous study of the PreRBP-TL [36]. These included the balanced accuracy (BACC), Matthew correlation coefficient (MCC), the area under the receiver-operating characteristic (ROC) curve (AUC), and the area under the precision-recall (PR) curve (AUPRC). BACC is a variant measure of accuracy, which is tailored for assessing the performance of a predictor on an imbalanced dataset [63]. MCC is an optimal metric for performance evaluation on the imbalanced dataset [64–66]. It measures the correlation coefficient between the actual labels and the predicted ones by considering the numbers of true positives, true negatives, false positives, and false negatives. BACC and MCC can be calculated as follows:

(7) \begin{equation*} \mathrm{BACC}=\frac{1}{2}\left(\frac{\mathrm{TP}}{\mathrm{TP}+\mathrm{FN}}+\frac{\mathrm{TN}}{\mathrm{TN}+\mathrm{FP}}\right) \end{equation*}

(8) \begin{equation*} \mathrm{MCC}=\frac{\mathrm{TP}\times \mathrm{TN}-\mathrm{FP}\times \mathrm{FN}}{\sqrt{\left(\mathrm{TP}+\mathrm{FP}\right)\left(\mathrm{TP}+\mathrm{FN}\right)\left(\mathrm{TN}+\mathrm{FP}\right)\left(\mathrm{TN}+\mathrm{FN}\right)}}, \end{equation*}

where *TP*, *TN*, *FP*, and *FN* represent the numbers of true positives, true negatives, false positives, and false negatives, respectively. The ROC curve plots the true positive rate (TPR) as the *y*-axis value and the false positive rate as the *x*-axis value. The PR curve plots precision on the *y*-axis and recall on the *x*-axis, describing the dynamic precision and recall changes in accordance with the prediction cutoff threshold. The values of AUC and AUPRC are positively related to the performance of predictors [67]. Because the datasets used in this study are highly imbalanced, the AUPRC metric was employed as the primary performance measure to select the optimized model based on the predictive performance evaluated on the validation sets [68].

## Results and discussion

### Validation of the framework design

In this section, we examined and validated the effectiveness of the designed RBP-TSTL in RBPs prediction by looking into the loss convergence during the model training process (Performance of the models on the annotated pre-training and species-specific datasets section). We assessed the RBPs prediction performance of different self-supervised pre-trained models. These models were used for generating the feature embeddings in Performance comparison of various self-supervised pre-trained models section. Next, we conducted the ablation study of the deep learning models in Ablation study section. All experiments in Validation of the framework design section were implemented on the training and validation sets, while the performance comparison in Sections Performance comparison of various self-supervised pre-trained models and Ablation study was performed on the validation sets and evaluated in terms of the AUPRC metric.

#### Performance of the models on the annotated pre-training and species-specific datasets

Transfer learning can effectively circumvent the scarcity of high-quality–labeled datasets [69]. It can improve the performance of target learners on target domains by transferring the knowledge from different but related source domains [70]. In deep transfer learning, a base network is first trained before its first *n* layers are copied to the corresponding layers of the target network. Next, the remaining layers of the target network are tuned for a target problem. There are two main strategies for training the target network: The first strategy is to back-propagate the loss in the entire target network to fine-tune it to the new problem, while the second one is to keep the transferred feature layers frozen, while the remaining layers are tuned to adapt to the target domain. Choosing whether to freeze the first *n* layers of the target network depends on the size of the target dataset and the distance between the source and target domains. If the dataset and distance between the source and target domains are small, tuning all layers may lead to over-fitting, and thus, the base layers should be kept frozen. On the other hand, if the target dataset is large and the distance between the source and target domains is considerably large, the over-fitting issue should not be a concern, and the base layers should be fine-tuned accordingly [71].

Here, we adopted the second strategy to implement transfer learning in view of the domain mismatch between the annotated pre-training and species-specific datasets. First, the base network was trained on the annotated pre-training RBPs dataset. Next, each species base network was tuned on the RBPs dataset to generate a species-specific model. We evaluated and compared the predictive performance of base and species-specific networks in this section. We contrasted the species-specific predictors with current state-of-the-art methods in Performance comparison between RBP-TSTL and other state-of-the-art methods section.

**Figure 2:**
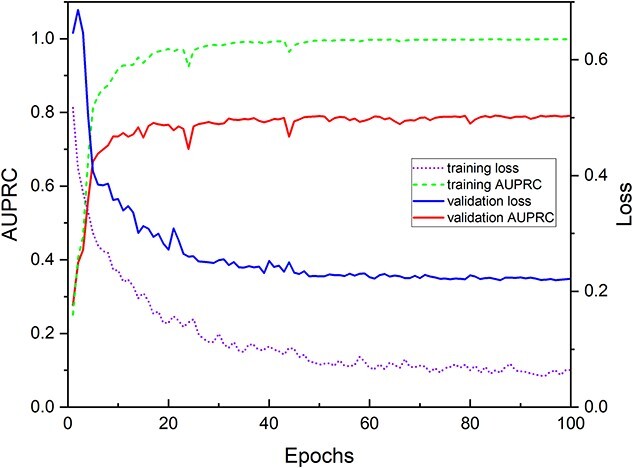
The average training loss and AUPRC of the base network trained on annotated pre-training RBPs dataset.

Figure 2 shows the changes in the average training loss and AUPRC, and Supplementary Fig. S1 (see Supplementary Data available online at https://academic.oup.com/bib) shows the changes in the average training accuracy and AUC for the baseline network trained on the annotated pre-training dataset over the consecutive training epochs. As can be seen, the training and validation loss of the network converged after about 60 epochs simultaneously, and the performance metrics for the validation set converged to a reasonably high value (i.e. ~0.8 for AUPRC, 0.95 for accuracy, and 0.95 for AUC, respectively, after about 60 epochs), while the performance metrics for the training set could approach one indefinitely. The results indicate that the base networks provided a high-quality non-species-specific prediction of RBPs and proved a strong foundation for the transfer learning of the species-specific predictors.

#### Performance comparison of various self-supervised pre-trained models

The effectiveness of the embeddings generated by the self-supervised pre-trained models can be attributed to two main factors: (i) language model (LM) architecture and (ii) the number of proteins used for pre-training (Table 2). The detailed introduction of various LMs can be found in the Supplementary Material. We chose ProtVec [18, 72], SeqVec [41], ProSE [73], ProtBert [43] and ProtT5-XL [43] as the representations of the three mainstream LM architecture, Word2Vec [74], Long-Short Term Memory (LSTM) [75], and Transformer [76]. To conduct the performance comparison, the embeddings generated by these LMs were inputted to the same deep learning model as used in RBP-TSTL. The performance of these models on validations sets was summarized in Table 3. We can see that ProtT5-XL achieved better performance than ProtBert on four validation sets, which indicates that LMs performance on RBPs prediction task is positively related to the size of the dataset that the LMs were trained on and the number of the model parameters. Additionally, ProSE performed better than SeqVec in spite of the similar model architecture. This suggests that the dataset with protein structural information on which the LM was trained is also helpful for boosting the model performance. With respect to the difference between the transformer-based and LSTM-based LMs, the self-attention mechanism used in the transformer-based models has proven to be more effective for capturing the long-range dependency [77]. In contrast, the model design of word2vec-based LMs can only map one word with its short-range context, as detailed in the Supplementary Material. In addition to the intrinsic feature of various LMs, the ProtT5-XL model has the most parameters and is pre-trained on the largest dataset, and the scalability of its performance in accordance with model size has been discussed in various empirical studies [78, 79]. As such, the embeddings generated by ProtT5-XL are more informative for predicting RBPs. Overall, ProtT5-XL, used in the RBP-TSTL for embeddings generation, achieved the best performance on the RBPs validation sets.

**Table 2:** Performance comparison of different self-supervised pre-trained models

Name	Model	Pre-trained proteins	Parameters	Dimensions
ProtVec [18, 66]	Word2Vec	530 K	<50 M	512
SeqVec [40]	LSTM	33 M	93 M	1280
ProSE [67]	LSTM	76 M unsupervised +28 K with structure information	~100 M	6165
ProtBert [42]	Transformer	216 M	420 M	1024
ProtT5-XL [42]	Transformer	261 M	3 B	1024

**Table 3:** Performance comparison of different models trained using different embeddings generated by protein LMs on the validation datasets

Lanugage model	Pre-training RBPs dataset	*H. sapiens*	*A. thaliana*	*E. coli*	*Salmonella*
ProtVec	0.24	0.43	0.35	0.53	0.74
SeqVec	0.53	0.75	0.62	0.81	0.97
ProSE	0.65	0.76	0.73	0.87	0.98
ProtBert	0.68	0.72	0.61	0.77	0.84
ProtT5-XL	0.79	0.80	0.85	0.97	1

*Note*: RBP-TSTL used ProtT5-XL for embeddings generation.

#### Ablation study

To investigate the impact of different components of the deep learning model used in the RBP-TSTL on the performance, an ablation study was conducted by modulating the number of GRU layers from 10 to 8, 6, 4, 2, and 0, respectively, and freezing the GRU layers during the fine-tuning process for six-layer-GRU network. The performance was evaluated on the validation sets. When the number of GRU layers equals zero, the network is reduced to a pure linear network. The more the number of GRU layers is, the more complex the network becomes. As shown in Table 4, RBP-TSTL achieved the most balanced performance on the annotated pre-training and four species-specific datasets, with the exception of the *E. coli* dataset where the zero-layer-GRU network achieved slightly better performance and on the pre-training dataset where the two-layer-GRU network achieved better performance. In addition, considering that larger deep learning models tend to favorably avoid under-fitting when a large pre-training dataset is involved [80], the results of the ablation study provide a reasonable justification for the use of a six-layer-GRU network to build the species-specific RBPs prediction models.

**Table 4:** Performance comparison of variant models with different numbers of GRU layers on the validation sets

Number of GRU layers	Pre-training RBPs dataset	*H. sapiens*	*A. thaliana*	*E. coli*	*Salmonella*
0	0.78	0.80	0.80	0.98	1
2	0.80	0.80	0.80	0.94	1
4	0.78	0.79	0.84	0.96	1
6	0.79	0.80	0.85	0.97	1
6 with freezing GRU layers	N/A	0.80	0.83	0.97	1
8	0.78	0.78	0.82	0.98	1
10	0.79	0.78	0.83	0.96	1

*Note*: RBP-TSTL used six layers of the GRU units.

### Impact of different transfer learning strategies on the model performance

We performed experiments to investigate the impact of different transfer learning strategies on model performance. The strategies examined include knowledge transfer across different species, either from the species in the annotated pre-training datasets to each of the four target species in the species-specific datasets detailed in Performance comparison of direct training, supervised pre-training, and general model with RBP-TSTL section or from a species to the other species within the four target species in Cross-species prediction of RBPs section. The performance evaluation in the experiments of this section was conducted on the independent test datasets.

#### Performance comparison of direct training, supervised pre-training, and general model with RBP-TSTL

**Figure 3:**
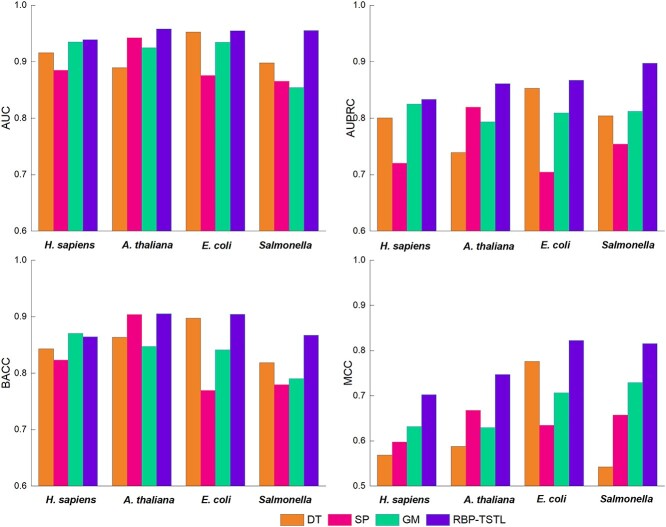
Performance comparison of various models on the four species-specific test sets. DT model was trained on four target species-specific datasets, SP model trained on the annotated RBPs dataset where four target species were excluded, and GM model trained on the mixture of all available datasets.

In this section, we conducted the performance comparison of several training and knowledge transfer strategies, including direct training (DT), supervised pre-training (SP), and general model (GM) with the RBP-TSTL training strategy. The DT models have only trained on each of the four respective target species-specific datasets, the SP models have trained on the annotated pre-training RBPs dataset, whereas the GM models were trained on the combined pre-training RBPs datasets and each of the species-specific datasets without the fine-tuning process. For the RBP-TSTL training strategy, the SP models were fine-tuned with each of the species-specific datasets. All these models are used as input the embeddings generated by the ProtT5-XL model in the same way as RBP-TSTL. The performance comparison results of these three models with RBP-TSTL are displayed in Fig. 3.

The results show that the RBP-TSTL models outperformed all other models for the RNA-binding protein prediction for all the four species in terms of the major performance metrics, e.g. AUC, AUPRC, and MCC (Fig. 3). The superior performance of RBP-TSTL indicates that its second-stage knowledge transfer could somehow help circumvent the issue of having limited annotated RBPs data. On the other hand, although the GM and RBP-TSTL models were trained on the same dataset, the domain clash between the annotated pre-training dataset and target datasets could introduce more noise into the models, thereby resulting in a reduced model performance of the GM models. In comparison, the RBP-TSTL models, which were further fine-tuned using the supervised pre-training based on each of the target species datasets, could effectively transfer the knowledge from the annotated pre-training RBPs dataset to the target species and avoid the domain clash between different species. As such, the RBP-TSTL model achieved a better performance than the other models.

#### Cross-species prediction of RBPs

**Figure 4:**
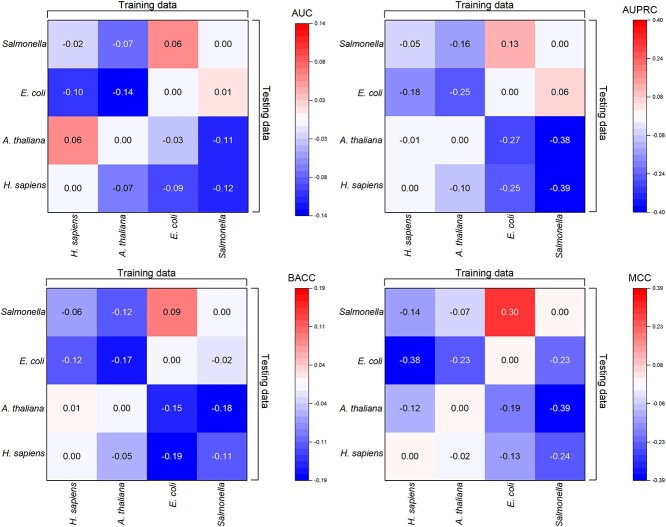
The results of cross-species prediction. The x-axis and y-axis represent the species of the RBPs for training and testing the predictor, respectively. The results of species-specific models are used as the baselines, and the heat maps show the increments of the cross-species model.

In this section, the cross-species prediction of RBPs was conducted to explore the evolutionary distance between different species. Figure 4 shows that the predictors trained on the *H. sapiens* dataset achieved similar performance on *A. thaliana* when being used as the species-specific predictor for *A. thaliana* and also vice versa. In contrast, the predictor trained on the *E. coli* dataset performed better on the *Salmonella* dataset in contrast to the species-specific predictor for *Salmonella*. Similarly, the predictor trained on the *Salmonella* dataset performed almost the same when being used as the species-specific predictor for *E. coli*. These are presumably because the numbers of sequences in the *H. sapiens* and *A. thaliana* datasets are similar, and these two species are evolutionarily close to each other. The results suggest that the RBPs predictors trained on the training datasets of *H. sapiens* and *A. thaliana* have nearly equivalent amount of knowledge with each other. In contrast, there are more sequences in the *E. coli* dataset than the *Salmonella* dataset and these two species are evolutionarily close. Therefore, the predictor trained on the *E. coli* dataset could potentially have more knowledge than the species-specific RBPs predictor trained on *Salmonella.* The results indicate that the sequences in the *H. sapiens* and *A. thaliana* datasets share high evolutionary similarities as is the same case for the *E. coli* and *Salmonella* datasets.

We also note that the results are in line with the fact that *H. sapiens* and *A. thaliana* are both eukaryotic organisms, while *E. coli* and *Salmonella* are prokaryotic organisms. In contrast to the cross-species prediction results in two previous works TriPepSVM [19] and PreRBP-TL [36], which suggested that the species-specific models performed overwhelmingly better than the cross-species ones, the performance of some cross-species models was found to be on par with that of the species-specific ones in the current study. This might be attributed to the fact that the embeddings generated by the self-supervised pre-trained model are not species-exclusive, and as such, the models trained on other species can potentially retain the predictive capability for the target species.

### Performance comparison between RBP-TSTL and other state-of-the-art methods

In this section, we conducted a comprehensive performance comparison between RBP-TSTL and the other state-of-the-art methods. Specifically, nine different sequence-based RBPs predictors were compared with RBP-TSTL on the independent test datasets. Afterward, we further compared three of the methods with RBP-TSTL on the additional validation datasets. Finally, three state-of-the-art feature encoding schemes were used to replace ProtT5-XL for generating the embeddings in the RBP-TSTL framework prior to the performance comparison with RBP-TSTL on the independent test datasets. The results are discussed in detail in the following subsections.

#### Performance comparison with nine state-of-the-art methods

We conducted the performance comparison between RBP-TSTL and nine sequence-based state-of-the-art methods on independent test datasets. As shown in Table 5, RBP-TSTL outperformed all other methods across the four species. This again illustrates the superiority of the two-stage transfer learning strategy for predicting the RBPs in both situations where the annotated data are relatively abundant (as in the case of *H. sapiens* and *A. thaliana*) or scarce (as in the case of *E. coli* and *Salmonella*). In addition, it is worth noting that the knowledge could also be transferred from the annotated pre-training RBPs datasets to each of the four target species in the PreRBP-TL [36]. However, a major difference between the two approaches is that the protein sequences were encoded by the PSSM features in PreRBP-TL. In summary, the superior performance of the proposed RBP-TSTL approach arises from two main factors: one is the highly informative embeddings generated by the self-supervised pre-trained LM and the other is the useful knowledge transferred from annotated pre-training RBPs dataset to the target species.

**Table 5:** Performance comparison of RBP-TSTL with nine existing methods on the independent test datasets of four species

Method^a^	*H. sapiens*				*A. thaliana*				*E. coli*				*Salmonella*			
	BACC	MCC	AUC	AUPRC	BACC	MCC	AUC	AUPRC	BACC	MCC	AUC	AUPRC	BACC	MCC	AUC	AUPRC
RNApred	0.66	0.22	0.72	0.27	0.73	0.36	0.86	0.35	0.65	0.20	0.75	0.37	0.72	0.35	0.79	0.46
SPOT-Seq-RNA	0.59	0.22	N/A	N/A	0.71	0.52	N/A	N/A	0.67	0.56	N/A	N/A	0.64	0.50	N/A	N/A
RBPPred	0.70	0.27	0.70	0.22	0.76	0.37	0.82	0.26	0.73	0.37	0.77	0.41	0.74	0.43	0.81	0.54
Deep-RBPPred^b^	0.63	0.19	0.70	0.28	0.67	0.18	0.75	0.22	0.70	0.26	0.72	0.27	0.68	0.28	0.69	0.27
Deep-RBPPred^c^	0.66	0.22	0.70	0.32	0.69	0.20	0.75	0.22	0.62	0.16	0.68	0.23	0.64	0.21	0.69	0.31
AIRBP	0.58	0.13	N/A	N/A	0.64	0.15	N/A	N/A	0.62	0.19	N/A	N/A	0.62	0.22	N/A	N/A
TriPepSVM^d^	0.69	0.36	0.79	0.41	0.72	0.36	0.82	0.28	0.73	0.37	0.83	0.47	0.71	0.38	0.79	0.50
TriPepSVM^e^	0.71	0.34	0.79	0.41	0.72	0.28	0.80	0.27	0.75	0.35	0.80	0.41	0.72	0.33	0.79	0.46
RBPro-RF^d^	0.71	0.32	0.79	0.37	0.75	0.28	0.82	0.33	0.71	0.33	0.76	0.32	0.76	0.43	0.81	0.60
RBPro-RF^e^	0.68	0.25	0.77	0.35	0.69	0.20	0.78	0.27	0.68	0.36	0.79	0.32	0.75	0.40	0.79	0.53
IDRBP-PPCT^d^	0.75	0.54	0.85	0.61	0.65	0.45	0.87	0.48	0.77	0.62	0.85	0.65	0.76	0.50	0.84	0.69
IDRBP-PPCT^e^	0.78	0.56	0.86	0.64	0.78	0.54	0.91	0.63	0.77	0.58	0.86	0.60	0.77	0.60	0.87	0.73
PreRBP-TL	0.80	0.61	0.89	0.72	0.86	0.71	0.95	0.80	0.86	0.72	0.93	0.81	**0.87**	0.72	0.95	0.86
RBP-TSTL	**0.87**	**0.69**	**0.94**	**0.82**	**0.91**	**0.75**	**0.96**	**0.86**	**0.90**	**0.82**	**0.95**	**0.87**	0.87	**0.82**	**0.96**	**0.90**

The highest values for each performance metric are bold.

^a^All results other than the one from RBP-TSTL were retrieved from [36]. The results of RNApred, SPOT-Seq-RNA, RBPPred, Deep-RBPPred, and AIRBP were calculated using their respective online web servers or stand-along packages. An in-house implementation generated the results of TriPepSVM, RBPro-RF, and IDRBP-PPCT.

^b^The results were calculated by using the stand-along package of Deep-RBPPred with a balanced model.

^c^The results were calculated by using the stand-along package of Deep-RBPPred with an imbalanced model.

^d^The results of the corresponding methods were calculated by the models trained with species-specific training data.

^e^The results of the corresponding methods were calculated by the models trained with the combination of pre-training data and species-specific training data.

Altogether, the results indicate that the self-supervised pre-trained model can be explored as a useful strategy for improving the performance of RBPs prediction.

#### Performance evaluation on the additional validation datasets

To evaluate the performance of RBP-TSTL in the practical scenario, we further tested the performance of RBP-TSTL on the additional validation datasets of experimentally validated *H. sapiens* RBPs. Here, we emphasized the TPRs as the primary performance measure as the additional validation datasets only include the positive samples. Accordingly, methods that have relatively lower performance but could present high sensitivity were not considered in the analysis. Therefore, it is of particular interest to compare the performance of RBP-TSTL with three other methods, i.e. TriPepSVM, IDRBP-PPCT, and PreRBP-TL on the additional validation sets. As shown in Table 6, RBP-TSTL was able to retrieve more RBPs than the other three methods in both set 1 and set 2. The results highlight the potential of RBP-TSTL for accurate prediction of RBPs.

**Table 6:** Performance comparison of RBP-TSTL and three existing method in terms of the TPRs for predicting *H. sapiens* RBPs on the additional validation datasets

Method	Validation Set 1	Validation Set 2
TriPepSVM^*^	75.45%	71.30%
IDRBP-PPCT^*^	69.09%	83.33%
PreRBP-TL	75.45%	92.59%
RBP-TSTL	81.82%	96.30%

All the results except for RBP-TSTL were retrieved from [36].

^a^The results were obtained using the corresponding in-house methods with a pre-training and species-specific training set.

#### Performance comparison of RBP-TSTL and the models trained with the same architecture but hand-crafted features

To further examine the performance of RBP-TSTL and better understand the impact of the embeddings generated by ProtT5-XL LM with respect to the knowledge transferred from the annotated pre-training dataset, we conducted the performance comparison between RBP-TSTL and models, for the latter of which, the state-of-the-art protein sequence encoding schemes were used to replace ProtT5-XL LM. Three types of encoding schemes were examined, including protein evolutionary features, physicochemical properties, and amino acid compositions. For the evolutionary features, we used PSI-BLAST [39] to search against the non-redundant (nr) database NRDB90 [81] to generate the PSSMs with three iteration and an e-value of 0.001. For those proteins whose PSSMs could not be generated by PSI-BLAST, the BLOSUM62 [36, 82] was used instead according to the previous study [83]. For the convenience of evolutionary information analysis, we converted the $L\times 20$ (*L* is the number of amino acids in the query protein sequence) PSSM profile generated from the previous procedure into a fixed dimensional vector. First, for each column, the values belonging to the same amino acid in all rows were summed to form a 20-dimensional vector. Then, the matrix was flattened to create a 20 × 20 = 400-dimensional vector [28].

For physicochemical information, the global composition feature encoding method (i.e. composition, transition, and distribution, C-T-D) [84–86] was utilized to encode the hydrophobicity, polarity, normalized van der Waals volume, polarizability, predicted secondary structure, and solvent accessibility. Additionally, the conjoint triad encoding method [87] was used to capture the charge and polarity information of the side chain in protein-RNA interaction. For the amino acid composition and sequence-order information, pseudo amino acid composition encoding mothed [88] was used. In this study, we used the iFeature package [89] to extract C-T-D, conjoin triad, and PACC features from primary protein sequences.

**Figure 5:**
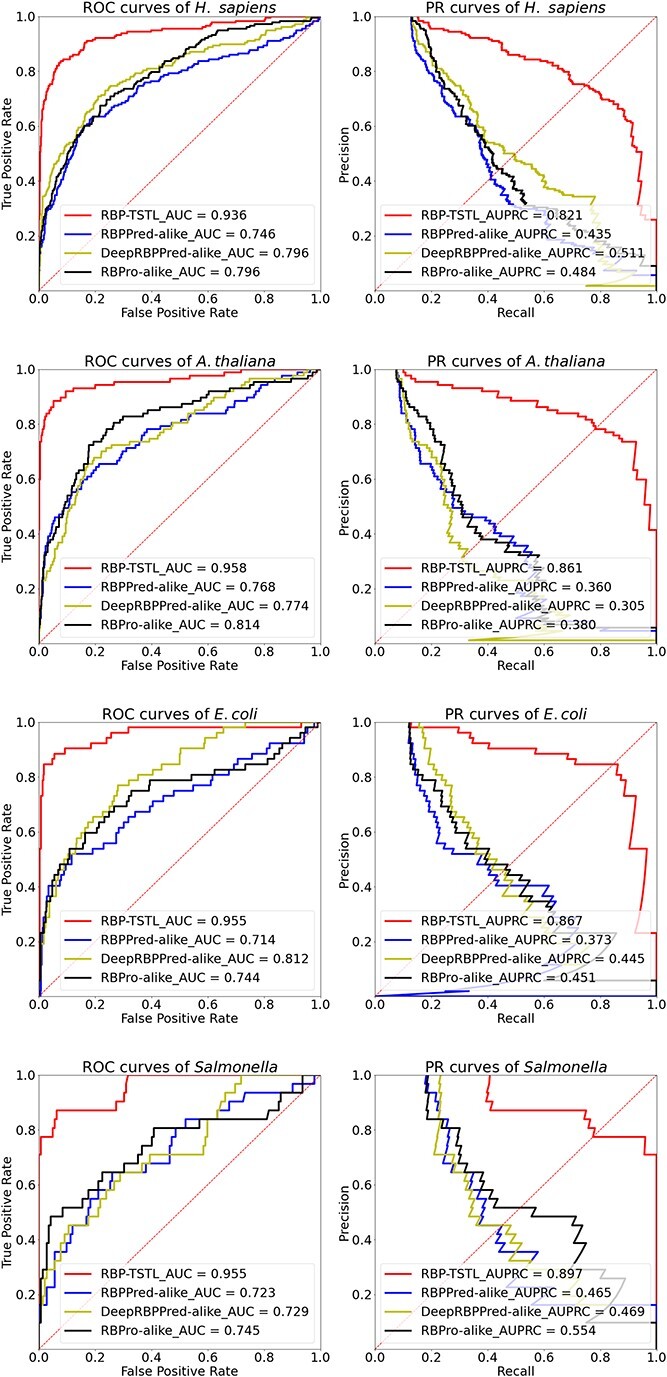
ROC and Precision-Recall curves of different methods on the independent test sets of four target species. RBPPred-alike, DeepRBPPred-alike, and RBPro-alike refer to the models built on the same encoding method as RBPPred, DeepRBPPred, and RBPro, respectively.

Three feature combinations were generated in accordance with the previous studies in recent years [17, 20, 21]. They included the PSSM-400 feature plus C-T-D plus conjoint triad, which followed the encoding methods in the RBPPred [21], C-T-D plus conjoint triad, which followed Deep-RBPPred [20], and PSSM-400 feature plus C-T-D plus PACC, which followed RBPro-RP [17]. These three encoding methods covered the major strategies to extract the hand-crafted features from the protein sequence by and large. Thus, we compared with RBP-TSTL the performance of these models trained with these features to further investigate the effectiveness of embeddings generated by LMs. As shown in Fig. 5, RBP-TSTL outperformed all the other models by a large margin across all the four species. The results highlight the value and importance of leveraging the self-supervised pre-trained model to further enhance the performance compared with the traditional encoding schemes.

### Genome-scale species-specific prediction of RBPs

In this section, we performed genome-scale species-specific prediction of putative RBPs of four target species by applying the best-performing RBP-TSTL model. The protein sequences of each species were downloaded from UniProt [50] using their corresponding taxonomy ID. In addition, protein sequences that appeared in previous training datasets were removed before the genome-wide screening. Protein sequences with more than 6000 amino acids in length were truncated due to the limitation of computational resources. At the final step, the calibration method [90] was employed to modulate the output logits to reflect the confidence level of the prediction. Finally, 17 935 protein sequences were predicted as the putative RBPs in *H. sapiens* with a confidence level of higher than 50%, 20 174 RBPs in *A. thaliana*, 144 686 RBPs in *E. coli*, and 222 192 RBPs in *Salmonella*, respectively. A statistical summary of the predicted RBPs with various confidence level in the four species is provided in Table 7. The genome-scale RBPs prediction results with different confidential levels are publicly available at https://github.com/Xinxinatg/RBP-TSTL, as a computational compendium for the wider research community to use.

**Table 7:** Statistical summary of the predicted RBPs with different confidence levels (CL) in *H. sapiens, A. thaliana, E. coli,* and *Salmonella*

Species CL	*H. sapiens*	*A. thaliana*	*E. coli*	*Salmonella*
*>*50%	17 935	20 174	144 686	222 192
*>*60%	15 851	18 857	132 930	203 636
*>*70%	13 958	17 559	121 281	183 896
*>*80%	12 207	16 366	110 553	160 762
*>*90%	10 067	14 599	96 872	133 240

## Conclusion and future work

In this study, we have designed and developed a new computational approach based on the pre-trained protein LM, termed RBP-TSTL, for high-performance, species-specific prediction of RNA-binding proteins at the genome-scale. Benchmarking experiments show that the proposed method outperformed several existing state-of-the-art methods in terms of the major performance metrics such as the AUC, AUPRC, and MCC. We conducted an extensive comparative analysis of different self-supervised pre-trained models for the RBPs prediction and proposed an effective two-stage transfer learning strategy. The first stage involved transferring knowledge from a self-supervised pre-trained model, while the second stage transferred the knowledge from the annotated non-species-specific RBPs dataset to each of the four target species. Performance benchmarking analysis showed that the species-specific RBP-TSTL models achieved state-of-the-art performance in RBPs prediction for *H. sapiens*, *A. thaliana*, *E. coli*, and *Salmonella*, despite the relative abundance or scarcity of the annotated data. We further applied the best-performing RBP-TSTL models and conducted the genome-scale prediction of RBPs in *H. sapiens*, *A. thaliana*, *E. coli*, and *Salmonella*. The predicted results are publicly available as a computational compendium at https://github.com/Xinxinatg/RBP-TSTL. We anticipate that the proposed RBP-TSTL approach will be leveraged as a useful tool and can inspire the development of self-supervised pre-trained protein LMs to explore their sequence–structure–function relationships.

Despite the excellent performance of RBP-TSTL for predicting RBPs, there exist several aspects that can be further improved by transferring the knowledge within eukaryotic or prokaryotic species and replacing the weighted binary cross-entropy methods with bootstrapping [91] to counter the data imbalance issue. As discussed in Cross-species prediction of RBPs section, the models of species within eukaryotes or prokaryotes could retain the predictive capabilities for cross-species RBPs prediction. Therefore, the target species with limited data could potentially benefit by transferring the knowledge from other species with more abundant data if they all belong to prokaryotes or eukaryotes. More specifically, the model of *A. thaliana* can be fine-tuned on top of the trained model of *H. sapiens*, and the model of *E. coli* can be fine-tuned on top of the trained model of *Salmonella*. Accordingly, the scarcity of data in certain species can be alleviated to some extent. In addition, the weighted binary cross-entropy might have some drawbacks in terms of counting the severe data imbalance. As an alternative approach, the bootstrapping method [45] can be used to tackle this data imbalance problem in the training dataset. In this way, the species-specific model for RBPs prediction can be potentially developed for the target species that have practically limited data.

**Key Points**

- Computational methods that are capable of accurately identifying RBPs are highly desirable and have important implications for biomedical and biotechnological applications.
- We designed and developed a new two-stage computational approach based on the pre-trained protein language model, termed RBP-TSTL, for high-performance, genome-scale, species-specific prediction of RNA-binding proteins.
- Benchmarking experiments showed that RBP-TSTL achieved better performance for predicting the RNA-binding proteins in *H. sapiens*, *A. thaliana*, *E. coli*, and *Salmonella* when compared with several existing methods.
- As a computational compendium, the predicted putative RBPs at the genome-scale are publicly accessible at https://github.com/Xinxinatg/RBP-TSTL for the research community to use.

## Supplementary Material

Supplementary_material_bbac215

## Data Availability

The source code of RBP-TSTL and genome-scale prediction results is publicly available at https://github.com/Xinxinatg/RBP-TSTL.

## Author Biographies

**Xinxin Peng** received his MS degree in Physics Science from La Trobe University in 2020. He is currently a research assistant in the Biomedicine Discovery Institute, Monash University and a member of the Bioinformatics and Computational Biomedicine Lab. His research interests include bioinformatics, computational biomedicine, machine learning and medical imaging.

**Xiaoyu Wang** received her master’s degree in information technology from The University of Melbourne in 2020. She is currently a research assistant in the Biomedicine Discovery Institute, Monash University and a member of the Bioinformatics and Computational Biomedicine Lab. Her research interests include bioinformatics, computational biomedicine and machine learning.

**Yuming Guo** is a professor of Global Environmental Health and Biostatistics & Head of the Monash Climate, Air Quality Research (CARE) Unit. His research focuses on environmental epidemiology, biostatistics, global environmental change, air pollution, climate change, urban design, residential environment, remote sensing modeling and infectious disease modeling.

**Zongyuan Ge** is an associate professor in the Monash e-Research Centre and Faculty of Engineering, Monash University. His research interests are artificial intelligence, computer vision, medical imaging, robotics and deep learning.

**Fuyi Li** received his PhD in Bioinformatics from Monash University, Australia. He is currently a Bioinformatics Research Officer in the Department of Microbiology and Immunology, Peter Doherty Institute for Infection and Immunity, The University of Melbourne, Australia. His research interests are bioinformatics, computational biology, machine learning and data mining.

**Xin Gao** is a professor of computer science in the Computer, Electrical and Mathematical Sciences and Engineering Division at the King Abdullah University of Science and Technology (KAUST), Saudi Arabia. He is also the Associate Director of the Computational Bioscience Research Center (CBRC), the Deputy Director of the Smart Health Initiative (SHI) and the Lead of the Structural and Functional Bioinformatics Group at KAUST. His research interests are bioinformatics, computational biology and machine learning.

**Jiangning Song** is an associate professor and group leader in the Monash Biomedicine Discovery Institute and the Department of Biochemistry and Molecular Biology, Monash University, Melbourne, Australia. He is also affiliated with the Monash Data Futures Institute, Monash University. His research interests include bioinformatics, computational biomedicine, machine learning and pattern recognition.
