# Supplementary material for: RBP-TSTL is a two-stage transfer learning framework for genome-scale prediction of RNA-binding proteins
Source: Brief Bioinform. 2022 Jun 2;23(4):bbac215. doi: 10.1093/bib/bbac215 (PMC9294422; doi:10.1093/bib/bbac215)
Supplement: Supplementary_material_bbac215 [file supplementary_material_bbac215.docx]

## Introduction of various pre-trained models

Staggering progress made in the natural language processing (NLP) [1-5] has motivated the bioinformatics community to study the application of NLP models on biological sequence-related problems. Because the sequence could essentially be seen as the language of life as it is contextual and vocabulary-based, it can also be used in pre-training practice to grasp the semantic information hidden in the primary sequences. There have been multiple pre-trained models for representation extraction from primary sequence. And different pre-training methods/model architectures would distill information of different latent spaces into the representation. For example, the feature representations generated from the pre-trained model purely through the self-supervision method [6] would potentially contain evolutionary information and the functional effects of sequence variations. And the pre-trained model with additional structure prediction tasks [7] would involve more specific protein structure information. In the following section, the different architecture of the pre-trained model will be discussed. The details of the models being used for the RBPs prediction task will be further given to elucidate the reason for the effectiveness.

**Word2Vec:**

Word2Vec [8] usually use two models to generate word embedding. One is the Continuous Bag of Words (CBOW) model, and another is the Skip-Gram model. For both models, the sentences are split into the context and the target. The context is defined by a window centered around the word to estimate, aka the target. For the CBOW model, the context is used as input to predict the target, whereas it is another way around for the Skip-Gram model.

The context and target are encoded with the one-hot encoding technique before being fed into the network. For CBOW, the output is to match with the target. In both models, the *i*-th weight of the hidden layer equals the embedding of each vocabulary according to their encoding position in the one-hot encoder.

The model we used here as an example of Word2Vec architecture is the reimplementation of ProtVec [9] in ProNA2020 [10] for the RBPs prediction. The model was trained using a skip-gram scheme on Swiss-Prot [11] as corpus, and the k-mer length is 3.

Word2vec usually maps one word with its short-range context or vice versa, while autoencoder could learn to map the entire contexts words with themselves. This enables the autoencoder to better grasp the contextual information. Following are the introduction of the Long-Short Term Memory (LSTM) [12] and Transformer [13], which are the two types of building blocks for autoencoders.

### LSTM

LSTM is a specially designed recurrent neural network to process sequential data with long-term dependencies. The core part of LSTM architecture includes the cell state that processes the sequential information, and the three gates are forgotten gate$f_{t}$, input gate $i_{t}$, and output gate $o_{t}$. They will decide whether to keep or forget the information from the previous state. The functions of these three gates are as follows: (1) Forget gate$f_{t}$ decides how much information needs to be abandoned in the cell state $c_{t-1}$ for the previous moment; (2) input gate $i_{t}$ controls how much information needs to be kept for the current cell state $c_{t}$; and (3) the output gate $o_{t}$ controls how much information of the current state$c_{t}$ shall be passed on to the external state.

$$f_{t}=\sigma\left( W_{f}\cdot\left[ h_{t-1},x_{t} \right]+b_{f} \right) (1)$$

$$i_{t}=\sigma\left( W_{i}\cdot\left[ h_{t-1},x_{t} \right]+b_{i} \right) \left( 2 \right)$$

$$\tilde{c}_{t}=tanh\left( W_{c}\cdot\left[ h_{t-1},x_{t} \right]+b_{c} \right) (3)$$

$$c_{t}=f_{t}\cdot c_{t-1}+i_{t}{\cdot\tilde{c}}_{t} (4)$$

$$o_{t}=\sigma\left( W_{o}\cdot\left[ h_{t-1},x_{t} \right]+b_{o} \right) (5)$$

$$h_{t}=o_{t}\cdot tanh(c_{t}) (6)$$

where $\sigma$ is the logistic function, $x_{t}$ the input at the current moment, and $h_{t-1}$ the external state from the previous moment, $W_{f}, W_{i}, W_{c}$ are the weight matrices for corresponding gate, and $b_{f}, b_{i}, b_{c}$ are the bias vectors.

In particular, the Embeddings from Language Model (ELMO) uses two layers of bidirectional LSTM (bi-LSTM) to get the contextual information [14], after a character-level convolutional neural network (CNN) convert words of a text string into raw word vectors. ELMO consist of two bi-LSTM, in this way, the protein sequences are input into the LSTM network, and during pre-training, given a sequence of N tokens, (t₁, t₂, …, tₙ), a forward language model infers the probability of the sequence by modeling the probability of token tₖ provided the history (t₁, …, tₖ₋₁). And similarly, the backward model computes the probability of token given the input of the tokens after the target one. The final representation is the weighted sum of the raw word vectors of the two intermediate word vectors. Therefore, the dataset in ELMO for self-supervised training could be established via masking out random words in a sentence, and the target is the word being masked. The input will be the context before and after this word.

SeqVec [15] and ProSE [7] were implemented on UniRef50 [16] dataset with ELMO architecture. Moreover, ProSE was trained with a multi-task strategy. The customized UniRef dataset was used for the self-supervised learning part and around 28,000 protein sequences from PDB with structures information for the supervised learning part.

### Transformer

Unlike the LSTM process data sequentially, Transformer can consider all inputs simultaneously and create a weight value for each word connection by attributing different weights to those inputs through the attention mechanism. The core part of the attention mechanism is applying the multiplication on the query (Q), keys (K), and value (V), which are all the vector representations of all the words in the sequence. Therefore, the contextual information will be included in the pairwise attention weight. The attention calculation is formulated as follows:

$$Attention(Q, K, V)=softmax(\frac{QK^{T}}{\sqrt{d_{k}}})V (7)$$

Here $d_{k}$ means the dimension of the key vector, and softmax means the softmax activation function.

The original Transformer is composed of Encoder and Decoder modules, including the self-attention mechanism. Meanwhile, the Decoder module has extra encoder-decoder attention to capture the relationship between embeddings generated from target and input. Because the attention mechanism attends to each of the individual tokens simultaneously, there is no prior knowledge of the relative position added to the input. The Transformer model manually adds the position information onto the input embeddings, which is the sinuous function of the position of the token.

In the Bidirectional Encoder Representations from Transformers (BERT) [17], only the Encoder module of the original Transformer architecture is used. In bidirectional language modeling, the model looks at all surrounding contexts of a masked token instead of just at the tokens preceding it. In this step, BERT first randomly masks out 15% of the vocabulary of the predicted text and train the model to make predictions based on the context. Afterward, these parameters are retained to continue training the model by identifying the continuity of the selected sentence. ProtBert [18] was trained on the UniRef100 dataset with the BERT architecture. ProtT5-XL [18] model followed the same strategy as BERT for denoising while retaining the main architecture of the original Transformer and was trained on the BFD dataset [19] before being fine-tuned on the UniRef50 dataset.

**Supplementary Table 1.** A summary of the tuned hyperparameter values

| Hyperparameters | Tuned setting |
| --- | --- |
| Batch size | 2048 |
| Initial learning rate | 0.00005 |
| Weight_decay | 0.0005 |
| ReduceLROnPlateau Scheduler patience | 5 |
| ReduceLROnPlateau Scheduler factor | 0.75 |
| Dropout rate | 0.05 |
| Activation function | LeakyReLU |


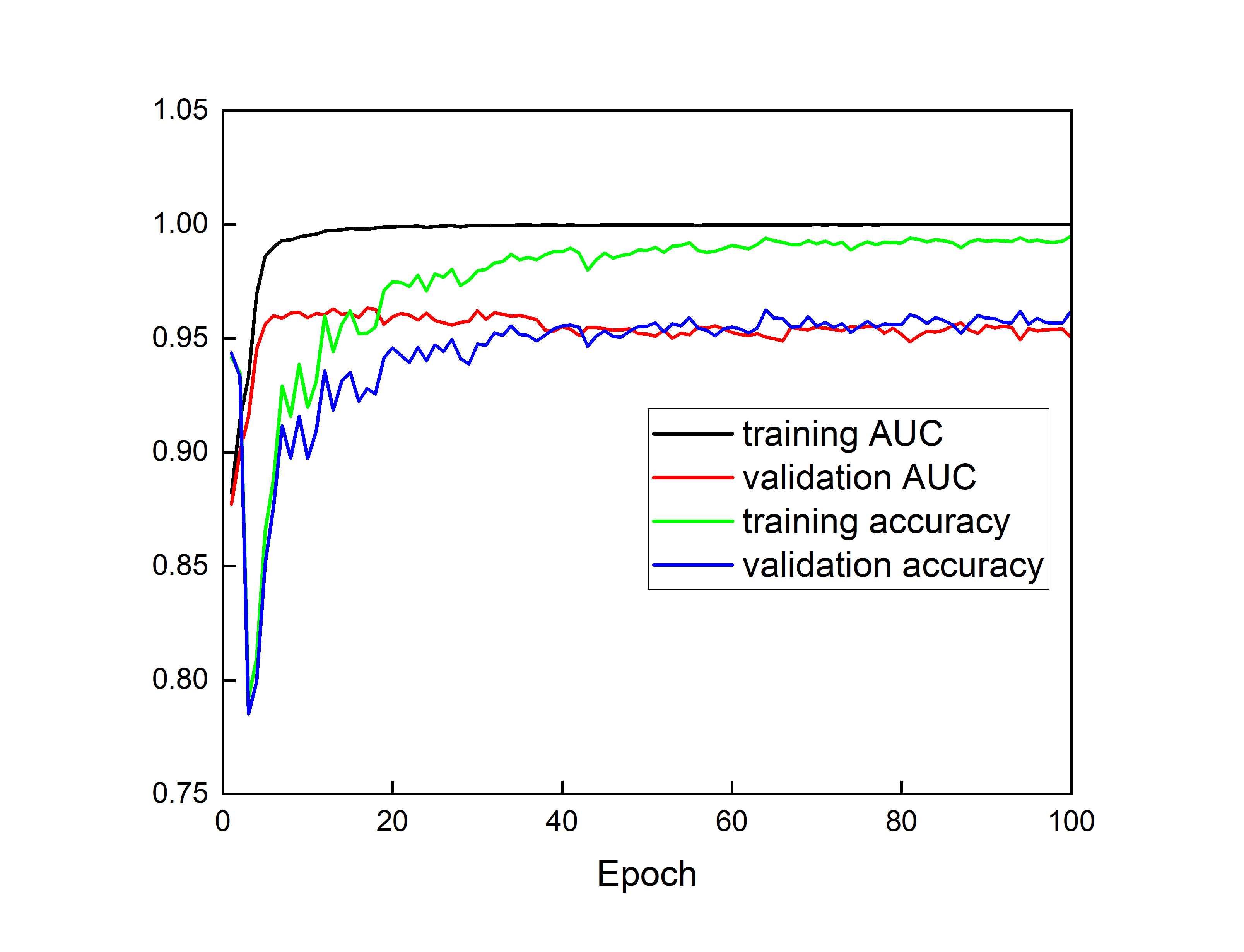


**Supplementary Figure S1.** The average training AUC and accuracy of the base network trained on annotated pre-training RBPs dataset.

## Experiment on whether hand-crafted features could further improve the performance of RBP-TSTL

In this section, we conducted an experiment to test whether hand-crafted features could further improve the performance of RBP-TSTL. The sequences were encoded by the concatenation of embeddings generated by ProtT5-XL and the hand-crafted features which comprised PSSM + C-T-D + PACC. The performance evaluation was conducted on independent testing sets. The results indicated that the hand-crafted features are not able to help improve the performance as shown in **Supplementary Table 2**. The reduced performance could arise from the fact that hand-crafted features are much less informative and noisier than the embeddings generated by the large-scale self-supervised pre-trained language model.

**Supplementary Table 2.** Comparison of performance between RBP-TSTL and model based on the concatenation of features generated by ProtT5-XLand hand-crafted features

| Method^a^ | *H. sapiens* | | | | *A. thaliana* | | | | *E. coli* | | | | *Salmonella* | | | |
| --- | --- | --- | --- | --- | --- | --- | --- | --- | --- | --- | --- | --- | --- | --- | --- | --- |
|  | **BACC** | **MCC** | **AUC** | **AUPRC** | **BACC** | **MCC** | **AUC** | **AUPRC** | **BACC** | **MCC** | **AUC** | **AUPRC** | **BACC** | **MCC** | **AUC** | **AUPRC** |
| RBP-TSTL | **0.87** | **0.69** | **0.94** | **0.82** | **0.91** | **0.75** | **0.96** | **0.86** | **0.90** | **0.82** | **0.95** | **0.87** | 0.87 | **0.82** | **0.96** | **0.90** |
| ProtT5-XL+hand-crafted features | 0.72 | 0.37 | 0.49 | 0.78 | 0.66 | 0.34 | 0.38 | 0.77 | 0.69 | 0.38 | 0.44 | 0.78 | 0.66 | 0.30 | 0.50 | 0.73 |

**References:**

1. Sun, L., et al., *Predicting dynamic cellular protein–RNA interactions by deep learning using in vivo RNA structures.* Cell Research, 2021. **31**(5): p. 495-516.

2. Ofer, D., N. Brandes, and M. Linial, *The language of proteins: NLP, machine learning & protein sequences.* Computational and Structural Biotechnology Journal, 2021.

3. Cui, F., Z. Zhang, and Q. Zou, *Sequence representation approaches for sequence-based protein prediction tasks that use deep learning.* Briefings in Functional Genomics, 2021. **20**(1): p. 61-73.

4. Song, B., et al., *Pretraining model for biological sequence data.* Briefings in Functional Genomics, 2021. **20**(3): p. 181-195.

5. Iuchi, H., et al., *Representation learning applications in biological sequence analysis.* Computational and Structural Biotechnology Journal, 2021. **19**: p. 3198.

6. Meier, J., et al., *Language models enable zero-shot prediction of the effects of mutations on protein function.* bioRxiv, 2021.

7. Bepler, T. and B. Berger, *Learning the protein language: Evolution, structure, and function.* Cell Systems, 2021. **12**(6): p. 654-669. e3.

8. Mikolov, T., et al., *Efficient estimation of word representations in vector space.* arXiv preprint arXiv:1301.3781, 2013.

9. Asgari, E. and M.R. Mofrad, *Continuous distributed representation of biological sequences for deep proteomics and genomics.* PloS one, 2015. **10**(11): p. e0141287.

10. Qiu, J., et al., *ProNA2020 predicts protein–DNA, protein–RNA, and protein–protein binding proteins and residues from sequence.* Journal of molecular biology, 2020. **432**(7): p. 2428-2443.

11. Boeckmann, B., et al., *The SWISS-PROT protein knowledgebase and its supplement TrEMBL in 2003.* Nucleic acids research, 2003. **31**(1): p. 365-370.

12. Hochreiter, S. and J. Schmidhuber, *Long short-term memory.* Neural computation, 1997. **9**(8): p. 1735-1780.

13. Vaswani, A., et al. *Attention is all you need*. in *Advances in neural information processing systems*. 2017.

14. Peters, M.E., et al., *Deep contextualized word representations.* arXiv preprint arXiv:1802.05365, 2018.

15. Heinzinger, M., et al., *Modeling aspects of the language of life through transfer-learning protein sequences.* BMC bioinformatics, 2019. **20**(1): p. 1-17.

16. Suzek, B.E., et al., *UniRef: comprehensive and non-redundant UniProt reference clusters.* Bioinformatics, 2007. **23**(10): p. 1282-1288.

17. Devlin, J., et al., *Bert: Pre-training of deep bidirectional transformers for language understanding.* arXiv preprint arXiv:1810.04805, 2018.

18. Elnaggar, A., et al., *ProtTrans: towards cracking the language of Life's code through self-supervised deep learning and high performance computing.* arXiv preprint arXiv:2007.06225, 2020.

19. Steinegger, M. and J. Söding, *Clustering huge protein sequence sets in linear time.* Nature communications, 2018. **9**(1): p. 1-8.
